# Supplementary material for: Density Functional Theory Study of Methanol Steam Reforming on Pt3Sn(111) and the Promotion Effect of a Surface Hydroxy Group
Source: Nanomaterials (Basel). 2024 Feb 5;14(3):318. doi: 10.3390/nano14030318 (PMC10857296; doi:10.3390/nano14030318)
Supplement: Supplementary file 1 [file nanomaterials-14-00318-s001.zip › nanomaterials-2846733-supplementary.pdf]

Supporting Information

Density Functional Theory Study of Methanol Steam Reforming on $Pt_3Sn(111)$ and the Promotion Effect of a Surface Hydroxy Group

Ping He [1,2], Houyu Zhu [2,*], Qianyao Sun [3], Ming Li [3], Dongyuan Liu [2], Rui Li [2], Xiaoqing Lu [2], Wen Zhao [2], Yuhua Chi [2], Hao Ren [2] and Wenyue Guo [1,2,*]

[1] College of Science, China University of Petroleum (East China), Qingdao 266580, China; s21090045@s.upc.edu.cn
[2] School of Materials Science and Engineering, China University of Petroleum (East China), Qingdao 266580, China; b21140013@s.upc.edu.cn (D.L.); s21140007@s.upc.edu.cn (R.L.); luxq@upc.edu.cn (X.L.); zhaowen@upc.edu.cn (W.Z.); chiyuhua@upc.edu.cn (Y.C.); renh@upc.edu.cn (H.R.)
[3] SINOPEC Dalian Research Institute of Petroleum and Petrochemicals Co. Ltd., Dalian 116045, China; sunqianyao.fshy@sinopec.com (Q.S.); liming.fshy@sinopec.com (M.L.)
* Correspondence: hyzhu@upc.edu.cn (H.Z.); wyguo@upc.edu.cn (W.G.)

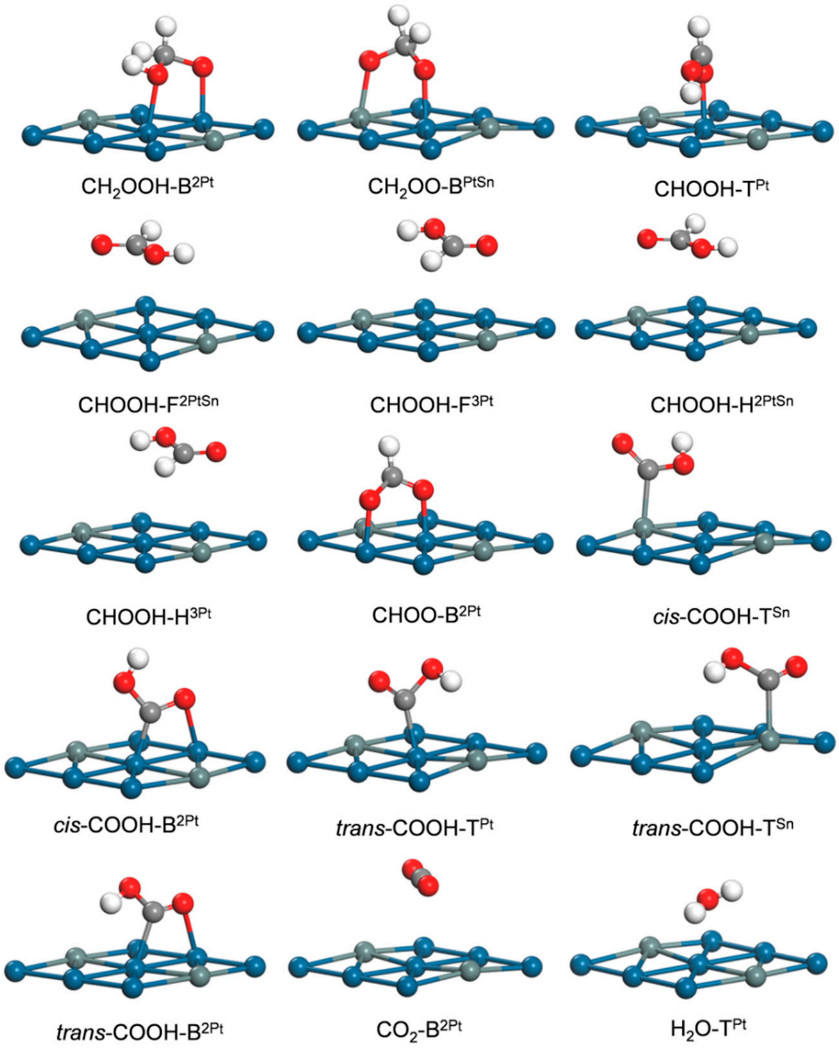

Figure S1. The other adsorption configurations of reaction intermediates along reaction pathway of Methanol Steam Reformation (MSR) to CO_2 on $Pt_3Sn(111)$.

Table S1. Sub-stable Adsorption Sites, Energies (in eV) and Structural Parameters (in Angstroms) for Intermediates Involved in MSR on $Pt_3Sn(111)$.

Species	Site[a]	Mode	$d_{C/O\text{-}Pt/Sn}$	E_{ads}
CH_2OOH	B^{2Pt}	η^1(O)-η^1(O)	2.14, 2.35	1.65
	B^{PtSn}	η^1(O)-η^1(O)	2.15, 2.31	1.89
CH_2OO	B^{PtSn}	η^1(O)-η^1(O)	2.05, 2.07	3.08
	F^{2PtSn}	η^2(O)-η^1(O)	2.09, 2.26, 2.27	3.24
HCOOH	T^{Pt}-V^b	η^1(O)	2.41	0.38
	T^{Sn}-V^b	η^1(O)	2.58	0.49
	F^{2PtSn}-P^b			0.41
	F^{3Pt}-P^b			0.36
	H^{2PtSn}-P^b			0.39
	H^{3Pt}-P^b			0.37
CHOO	B^{2Pt}	η^1(O)-η^1(O)	2.20, 2.20	2.28
	B^{PtSn}	η^1(O)-η^1(O)	2.17, 2.29	2.52
COOH-*cis*	T^{Pt}	η^1(C)	2.03	2.48
	T^{Sn}	η^1(C)	2.32	1.26
	B^{2Pt}	η^1(C)-η^1(O)	2.01, 2.36	2.37
COOH-*trans*	T^{Pt}	η^1(C)	2.06	2.35
	T^{Sn}	η^1(C)	2.34	1.09
	B^{2Pt}	η^1(C)-η^1(O)	2.02, 2.37	2.39
	B^{PtSn}	η^1(C)-η^1(O)	2.04, 2.55	2.41
CO_2	B^{2Pt}			0.11
	B^{PtSn}			0.11
H_2O	T^{Pt}			0.01
	T^{Sn}			0.01
OH	T^{Pt}	η^1(O)	2.04	2.34
	T^{Sn}	η^1(O)	2.04	2.50
	B^{2Pt}	η^2(O)	2.23, 2.24	2.51
	B^{PtSn}	η^2(O)	2.25, 2.27	2.49

[a] V and P represent the O–H axis almost vertical and parallel to the surface.
